# CircUBE2D2 regulates HMGB1 through miR-885-5p to promote ovarian cancer malignancy

**DOI:** 10.1016/j.clinsp.2024.100391

**Published:** 2024-06-06

**Authors:** RuiXue Yan, SaiTian Zeng, FangYuan Gao, LingLing Li, XiYun Xiao

**Affiliations:** Department of Gynecology I, Cangzhou Central Hospital, Cangzhou City, Hebei Province, China

**Keywords:** Ovarian Cancer, CircUBE2D2, miR-885-5p, ceRNA, HMGB1

## Abstract

**Background:**

The newly discovered CircUBE2D2 has been shown to abnormally upregulate and promote cancer progression in a variety of cancers. The present study explored circUBE2D2 (hsa_circ_0005728) in Ovarian Cancer (OC) progression.

**Methods:**

CircUBE2D2, miR-885-5p, and HMGB1 were examined by RT-qPCR or WB. SKOV-3 cell functions (including cell viability, apoptosis, migration, and invasion) were validated using the CCK-8, flow cytometry, scratch assay, and transwell assay, respectively. The direct relationship between miR-885-5p and circUBE2D2 or HMGB1 was confirmed by a dual-luciferase reporter and RNA pull-down analysis. circUBE2D2′s role in vivo tumor xenograft experiment was further probed.

**Results:**

OC tissue and cell lines had higher circUBE2D2 and HMGB1 and lower miR-885-5p. Mechanically, CircUBE2D2 shared a binding relation with miR-885-5p, while miR-885-5p can directly target HMGB1. Eliminating circUBE2D2 or miR-885-5p induction inhibited OC cell activities. However, these functions were relieved by down-regulating miR-885-5p or HMGB1 induction. Furthermore, circUBE2D2 knockout reduced tumor growth.

**Conclusion:**

CircUBE2D2 regulates the expression of HMGB1 by acting as a sponge of ceRNA as miR-885-5p, thereby promoting the control of OC cell proliferation and migration and inhibiting cell apoptosis. Targeting CircUBE2D2 could serve as a new potential treatment strategy for OC.

## Highlights

• CircUBE2D2 subcellular localization and RNAse R resistance analysis.
• Expression patterns of circUBE2D2 and miR-885-5p.
• CircUBE2D2 silencing inhibits SKOV-3 cell malignant phenotypes.
• CircUBE2D2 serves as the ceRNA of miR-885-5p.

## Introduction

Ovarian Cancer (OC) is highly invasive and metastatic, among which high-grade serous carcinoma originating from the ovarian epithelium is the most common.^1^ The staging of OC is determined by the International Federation of Gynecologists and Obstetricians.^2^ Most epithelial OC cases are diagnosed as advanced. However, despite aggressive surgery and chemotherapy or immunotherapy, the survival rate of patients with advanced epithelial OC remains low, and more effective diagnostic and therapeutic approaches are urgently needed.^3^

CircRNAs are highly conserved and stable noncoding RNA molecules with a unique covalent closed-loop structure.^4^ The production and function of circular RNA remain incompletely elucidated in the academic literature; however, it has been demonstrated to impact tumor development through diverse mechanisms. Of these mechanisms, circular RNA's role as a microRNA (miRNA) sponge is considered paramount, as it can modulate gene expression by competitively binding to miRNA molecules as a competitive endogenous RNA (ceRNA).^5^ Abnormally expressed circRNAs in OC are related to its diagnosis or prognosis ^6^ and are related to OC progression.^7^^,^^8^ It is elaborated that circUBE2D2 regulates CDca_3 through miR-512-3p to induce mesothelioma progression.^9^ Whether circUBE2D2 mediates OC cell activities still lacks an in-depth study. miRNAs are tiny noncoding RNAs composed of only 17‒22 nucleotides ^10^ that can bind to 3’UTRs and alter downstream target expression by mRNA degradation induction or translation inhibition.^11^ Some miRNAs have been identified to be associated with OC, for example, miR-425-5p and miR-760 promote OC cell proliferation and metastasis,^12^^,^^13^ and miR-143, miR-365 and miR-4324 inhibit metastasis.^14^, ^15^, ^16^ It is known that miR-885-5p can inhibit tumor cell proliferation and metastasis.^17^, ^18^, ^19^ HMGB1 has been measured to be highly expressed in tumor cells 20, 21, 22 and is a new potential OC marker, especially for epithelial OC.^23^ Therefore, the authors hypothesize that CircUBE2D2 may act as ceRNA to regulate the miR-8851-5p/HMGA1 axis, thereby participating in the regulation of OC progression.

The main objective of this study was to investigate the effects of a novel circular RNA, CircUBE2D2, on the biological function of OC cells. Various experimental methods and bioinformatics software were used to explore the interaction between CircUBE2D2 and miR-885-5p and HMGB1, as well as how CircUBE2D2 promotes the occurrence and development of OC cells by regulating the miR-885-5p/HMGB1 axis to provide new insights and therapeutic prospects into the regulatory mechanisms of CircUBE2D2 in OC.

## Materials and methods

### Clinical tissue sample

30 patients with OC who underwent surgery were enrolled, all of whom did not receive any preoperative local or systemic treatment. Table 1 demonstrates the patient's clinical features. Cancer tissues and para-cancer non-tumor tissues were collected, frozen in liquid nitrogen, and stored at -80 °C for the convenience of subsequent experimental studies. This human study was approved by the Ethics Committee of Cangzhou Central Hospital (Approval nº 202006CZ37) Consent was obtained from all patients or family members and written informed consent was signed prior to surgery.

**Table 1.** Clinical characteristics of patients.

Characteristics		Number	Percentage (%)
**Age**	<50	12	40
	≥ 50	18	60
**Histotype**	Serous	15	50
	Endometrioid	8	26.67
	Mucinous	5	16.67
	Clear cell	2	6.67
**Tumor differentiation**	Low	18	60
	Medium‒High	12	40
**Lymph node metastasis**	Yes	13	43.33
	No	17	56.67

### Cell line and culture

Normal ovarian epithelial cells HOECs and human OC cell lines SKOV-3, HO-8910, A2780 and OVCAR3 cells were derived from Shanghai Institutes for Biological Sciences. All cells were cultured in RPMI 1640 or DMEM containing 10 % FBS (Gbico), 100 U/mL penicillin, and 100 μg/mL streptomycin (Sigma-Aldrich) and placed in a 5 % carbon dioxide humidified incubator at 37 °C.

### Cell transfection

GenePharma (Shanghai, China) provided oligonucleotides and plasmids. For studies of circUBE2D2 knockdown, SKOV-3 cells with 50 % confluence were transiently transfected with 20 nM siRNA for circUBE2D2 (si-CircUBE2D) or a disrupted Negative Control (si-NC). For induction studies, 100 ng pcDNA3.1 based HMGB1 induction plasmid (HMGB1) or negative pcDNA control was transfected. miR-885-5p mimics (20 nM), miR-885-5p inhibitors (20 nM), or negative controls (mimic-NC or inhibitor-NC) produced miR-885-5p overexpressed or knockdown cells. The culture medium was replaced 6 h after transfection, and 48 h after transfection, transfection efficiency in cells was evaluated by RT-qPCR, and follow-up experiments were performed. For the *in vivo* silencing study for circUBE2D2, 50 % confluent SKOV-3 cells were infected with shRNA lentivirus for circUBE2D2 (sh-CircUBE2D2) or negative controls for 24 h, and then virus-mediated cells were treated with 1 μg/mL puromycin for 14 days.

### RNA isolation and RT-qPCR

MolPure® Cell/Tissue Total RNA Kit (YEASEN, Shanghai, China) extracted total RNA from tissues or cultured cells. Assessments of RNA concentration and purity were completed with a NanoDrop 2000 spectrophotometer (Thermo Fisher Scientific). Random primers and stem ring primers of Oligo (dT)18 or miR-885-5p were added to synthesize cDNA by reverse transcription based on Hifair® Ⅱ 1^st^ Strand cDNA Synthesis Kit (gDNA digester plus) (YEASEN). RT-qPCR started with Hieff®qPCR SYBR®Green Master Mix Kit (YEASEN) in LightCycler 480 real-time PCR instrument (Roche, Basel, Switzerland). RNAs were calculated by 2^−△△Ct^, circRNA, and mRNA were referenced by GAPDH, and miRNA was by U6. All primers for RT-qPCR are listed in Table 2.

**Table 2.** Primer Sequence.

Gene	Forward Primer	Reverse Primer
CircUBE2D2	GATCACAGTGGTCTCCAGCA	GCCCCATTATTGTAGCTTGC
miR-885-5p	RT:GTCGTATCCAGTGCAGGGTCCGAGGTATTCGCACTGGATACGACAGAGGC	
	GCGCGTCCATTACACTACCCT	AGTGCAGGGTCCGAGGTATT
HMGB1	TATGGCAAAAGCGGACAAGG	CTTCGCAACATCACCAATGGA
GAPDH	CACCCACTCCTCCACCTTTG	CCACCACCCTGTTGCTGTAG
U6	CTCGCTTCGGCAGCACA	AACGCTTCACGAATTTGCGT

### CircUBE2D2 RNAse R resistance analysis and subcellular mapping

Total RNA isolated from cells was incubated at 37 °C for 30 min with or without 4 U/mg RNAseR (GENESEED, Guangzhou, China). CircUBE2D2 and UBE2D2 mRNA were detected by RT-qPCR, and the resistance of CircUBE2D2 to RNAse R was evaluated.

For subcellular localization analysis, RNA from the nucleus and cytoplasm of 1 × 10^6^ SKOV-3 cells was isolated using the PARIS kit (Life Technologies, USA). Gene expression was assessed by RT-qPCR, taking GAPDH and U6 as the cytoplasmic and nuclear references, respectively.

### Western blotting

The cells and tissues were washed with pre-cooled PBS, and lysis buffer (Beyotime, Shanghai, China) was added and placed on ice for lysis for 20 min. Bradford assay (Bio-Rad, USA) analyzed protein concentration. The proteins were then isolated by 15 % SDS-PAGE and loaded to PVDF membranes. At room temperature, they were sealed with 5 % skim milk powder for 1 h, then washed three times with TBST, and incubated overnight with primary antibody HMGB1 (ab228624, Abcam, UA) and β-actin (ab115777, Abcam) at 4 °C. The secondary antibody (CST, USA) bound to horseradish peroxidase was incubated at 37 °C for 1 h and developed using an ECL kit (ultrassignal, China).

### CCK-8 detection

Cell viability assay was conducted using the CCK-8 detection (Dojindo, Japan) at 0 h, 24 h, 48 h, and 72 h after transfection. In the 96-well plates, 2 × 10^3^ SKOV-3 cells were added in each well and incubated with 10 µL CCK-8 reagent at each time point. The plates required 2 h incubation before reading the absorbance at 450 nm on a microplate reader (Bio-Rad).

### Flow cytometry

Apoptosis rates were assessed by the FITC-Annexin V Apoptosis Detection Kit (BD Biosciences). At 48 h after transfection, SKOV-3 cells were washed twice with pre-cooled PBS and re-suspended in 500 μL 1 × binding buffer. Next, 5 μL Annexin V-FITC and 5 μL PI solutions were incubated together. After 15 min, the proportion of apoptotic cells was measured on a FACScan flow cytometer (BD Biosciences).

The cell cycle was analyzed using PI staining for DNA content. Simply put, SKOV-3 cells were fixed overnight with cold 70 % ethanol for PI staining at 20 μg/mL. Flow cytomety (BD Bioscience) was conducted for analysis of the cell cycle after 30 min of protection from light.

### Scratch assay

At 48 h after transfection, SKOV-3 cells were made into a suspension. The suspension was mixed with DMEM without FBS, of which the concentration was adjusted to 5 × 10^6^ cells /mL. With 80 % confluence, cells were wounded by cross-shaped scratches with 1 mL gun tip. Cell mass was washed away with PBS, and the culture medium was changed. At 0 h, 24 h, and 48 h after the scratch, the scratch width was observed.

### Invasion analysis

Transwell chambers (BD Biosciences) were coated with matrix gel (BD Biosciences), of which the upper compartment contained 200 μL cell suspension (5 × 10^6^ cells/mL), and the lower compartment contained 500 μL DMEM + 20 % FBS. After 24 h, the remaining migratory and invasive cells were fixed with 4 % paraformaldehyde and stained with 0.5 % crystal violet. Cell images were taken under an inverted microscope (Olympus) in visual fields.

### Luciferase activity detection

StarBase 3.0 (http://starbase.sysu.edu.cn/) predicted the binding site between miR-885-5p 3′UTR segment and CircUBE2D2. TargetScan 3.0 7.1 (http://www.targetscan.org/vert_71/) and starBase predict miR-885-5p targets. miR-885-5p binding sites-contained wild type and mutant circUBE2D2 and HMGB1 3ʹUTR sequences were inserted into the pmirGLO carrier. The luciferase reporter was co-transfected into the cells with miR-885-5p mimics or control mimics using Lipofectamine® 2000. After 48 h, each well of 96-well plates was supplementary with 100 μL lysis buffer and centrifuged at 10,000‒15,000 g for 3‒5 min to measure luciferase activities based on the dual luciferase reporter assay kit (Beyotime).

### RNA pull-down and RIP analysis

Cell lysate products were collected with RIPA lysis buffer. For RNA pull-down, the product was combined with Bio-miR-885-5p or Bio-miR-NC (GenePharma) and co-incubated with streptavidin magnetic beads (Thermo Fisher Scientific) at 4 °C. CircUBE2D2 in purified RNA was analyzed by RT-qPCR.

### Animal research

All animal studies were conducted in accordance with protocols approved by the Cangzhou Central Hospital Animal Care and Use Committee (Approval No. 202019CZ39). Twelve 6-week-old BALB/c nude mice (Shanghai Laboratory Animal Research Center) were implanted subcutaneously with 5 × 10^6^ SKOV-3 cells transfected with sh-circUBE2D2 or sh-NC at the left abdomen. Tumor volume = 1/2 (length × width^2^). Euthanasia was executed on day 27 after implantation.

### Data analysis

G-power 3.1 (University of Düsseldorf, Düsseldorf, Germany) was utilized for sample size calculation and power analysis in this study. A one-way analysis of variance was conducted to compare the levels of CircUBE2D2 between the tumor and non-tumor groups. Based on the difference in means between the two groups, with a significance level (α) of 0.05 and a test power (1-β) of 0.8, it was determined that the power of the test was 80 % and the minimum required sample size was 24. The results are presented as mean ± standard deviation. Statistical differences were evaluated using one-way ANOVA and post hoc Tukey Kramer test with the assistance of data analysis software (SPSS 19.0, SPSS Inc., Chicago, IL, USA); p < 0.05 was considered statistically significant.

## Results

### CircUBE2D2 subcellular localization and RNAse R resistance analysis

**Fig. 1:**
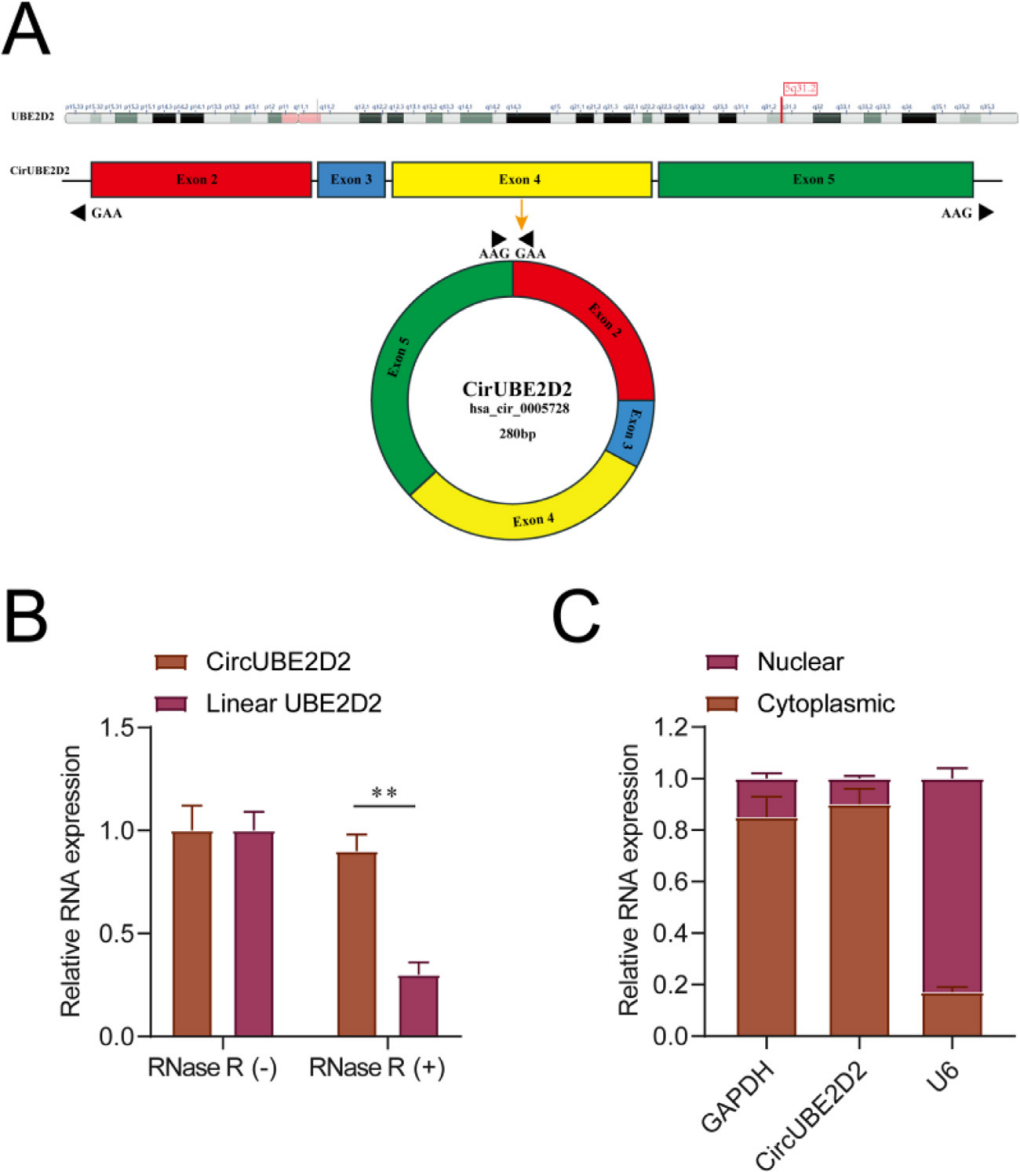
CircUBE2D2 subcellular localization and RNAse R resistance analysis. (A) CircUBE2D2 structure. (B) RT-qPCR measurements of circular and linear UBE2D2 mRNA with or without RNAse R. (C) RT-qPCR measurements of circUBE2D2 in the nucleus and cytoplasm (*p < 0.05).

In the CircBase, circUBE2D2 (hsa_circ_0005728) is derived from exons 3 through 5 of the UBE2D2 gene on chromosome 5q31.2 by folding back, with a length of 280 bp (Fig. 1A). Subsequently, linear UBE2D2 was found to be degraded by RNAse R, but circUBE2D2 was not digested by RNAse R (Fig. 1B). RT-qPCR results from nuclear and cytoplasmic components showed that circUBE2D2 was primarily located in the cytoplasm of SKOV-3 cells (Fig. 1C).

### Expression patterns of circUBE2D2 and miR-885-5p

**Fig. 2:**
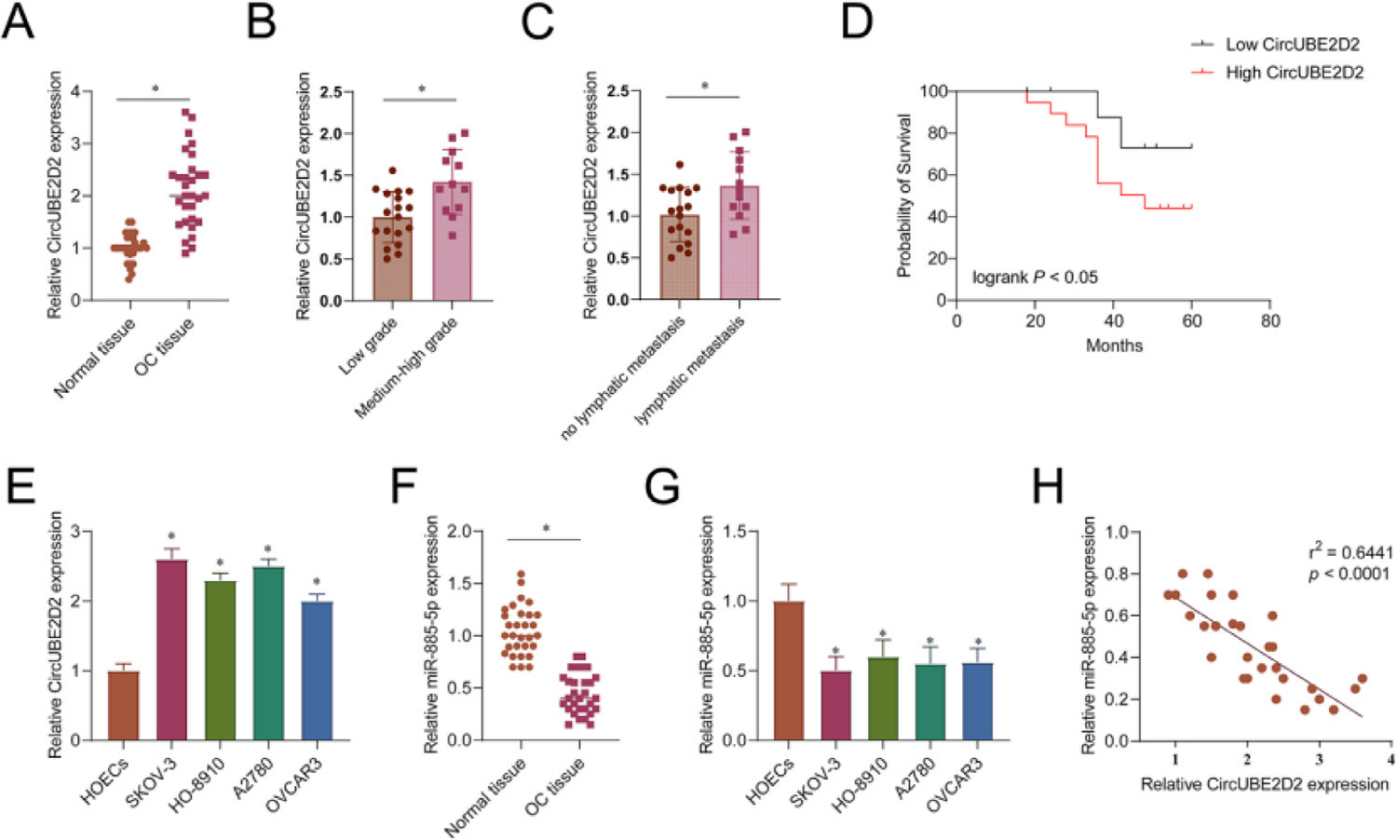
Expression signatures of circUBE2D2 and miR-885-5p. (A) RT-qPCR measurements of CircUBE2D2 in OC tissue and matched normal tissue. (B) Relative expression of CircUBE2D2 in tumor tissues with different degrees of differentiation. (C) Relative expression of CircUBE2D2 in tumor tissue with or without lymphatic metastasis. (D) The median value of CircUBE2D2 was set as the cut-off value, and the Kaplan-Meier method was used to determine the correlation between the expression of CircUBE2D2 and the overall survival of OC patients. (E) The relative expression of CircUBE2D2 in normal Ovarian Epithelial Cells (HOECs) and OC cells (SKOV-3, HO-8910, A2780 and OVCAR3). (F) Relative expression of miR-885-5p in OC tissues and normal tissues. (G) Relative expression of miR-885-5p in normal ovarian epithelial cells and OC cells. (H) Spearman test to study the correlation between miR-885-5p expression and circUBE2D24 level in OC (*p < 0.05).

CircUBE2D2 and miR-885-5p levels in OC tissues and cells were detected by RT-qPCR. CircUBE2D2 in OC was increased (Fig. 2A). An increase in CircUBE2D2 was observed in highly differentiated tumor tissues compared to poorly differentiated tumor tissues (Fig. 2B). CircUBE2D2 expression was upregulated in tumor tissues with lymphatic metastasis compared with those without lymphatic metastasis. Kaplan-Meier survival curve analysis showed that OC patients with higher CircUBE2D2 expression had poorer overall survival (Fig. 2C). In addition, the OC cell line showed a higher level of circUBE2D2 than normal HOECs (Fig. 2D). Thus, abnormal CircUBE2D2 expression is associated with the progression and prognosis of OC. In contrast, miR-885-5p was lower in OC tissues and cell lines (Fig. 2E‒F). A strong negative correlation was found between miR-885-5p and circUBE2D2 expression in OC tissues (Fig. 2G).

### CircUBE2D2 silencing inhibits SKOV-3 cell malignant phenotypes

**Fig. 3:**
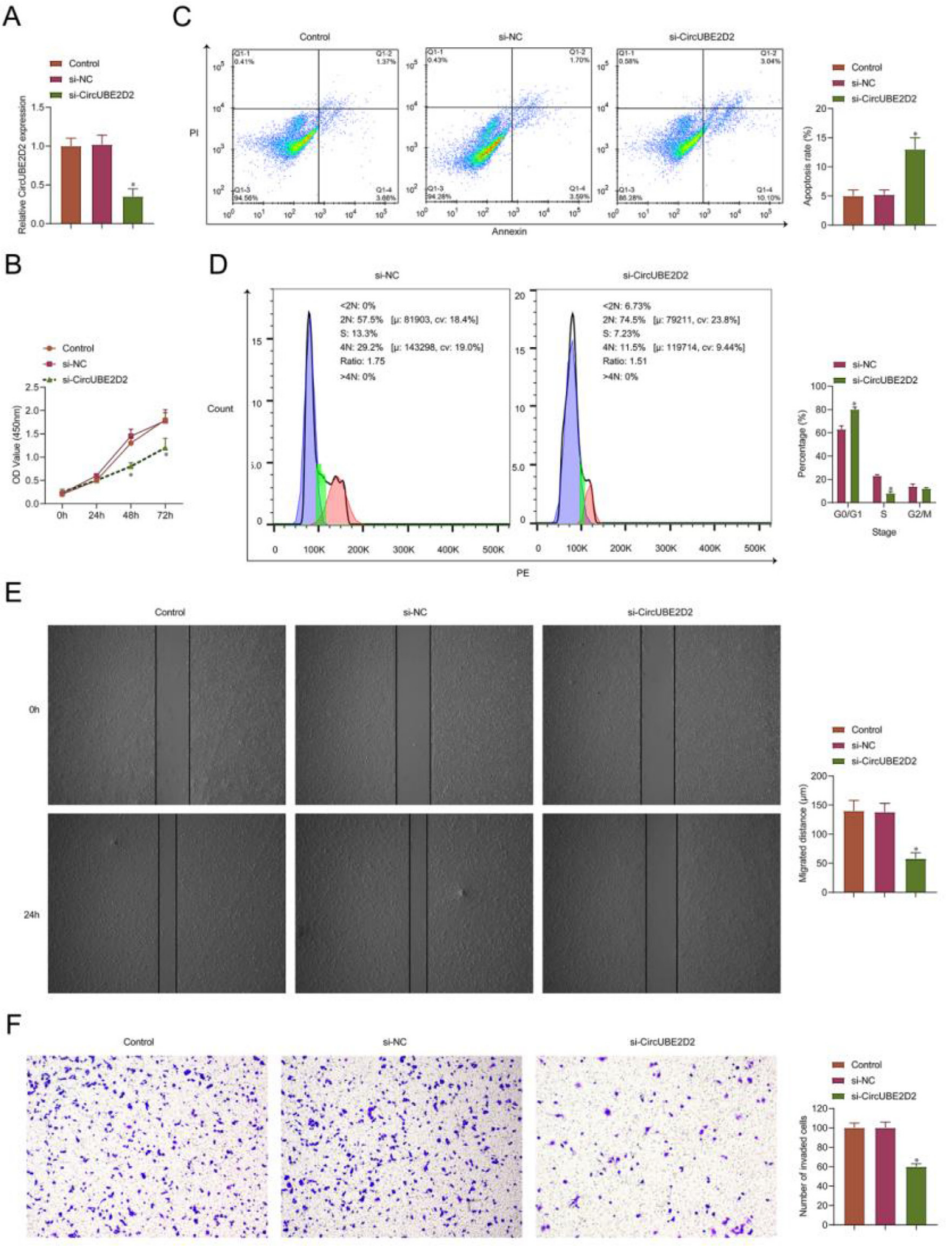
CircUBE2D2 silencing inhibits SKOV-3 cell malignant phenotypes. SKOV-3 cells were transfected with si-NC or si-CircUBE2D2. (A) RT-qPCR measurements of CircUBE2D2. (B) Determination of CCK-8 cell viability. (C‒D) Flow cytometry analysis of apoptosis and cell cycle. (E) Cell scratch migration assay. (F) Assay of Transwell invasion (×100, *p < 0.05).

The authors performed a loss-function analysis concerning circUBE2D2 *in vitro*. Transient transfection of si-circUBE2D2 resulted in reduced expression of circUBE2D2 (Fig. 3A). In SKOV-3 cells, circUBE2D2 knockdown repressed cell viability (Fig. 3B) and induced apoptosis (Fig. 3C). Meanwhile, knockdown of circUBE2D2 also increased the proportion of SKOV-3 cells in the G0/G1 phase (Fig. 3D). In addition, the absence of circUBE2D2 inhibited cell migratory (Fig. 3E) and invasive (Fig. 3F) rates.

### CircUBE2D2 serves as the ceRNA of miR-885-5p

**Fig. 4:**
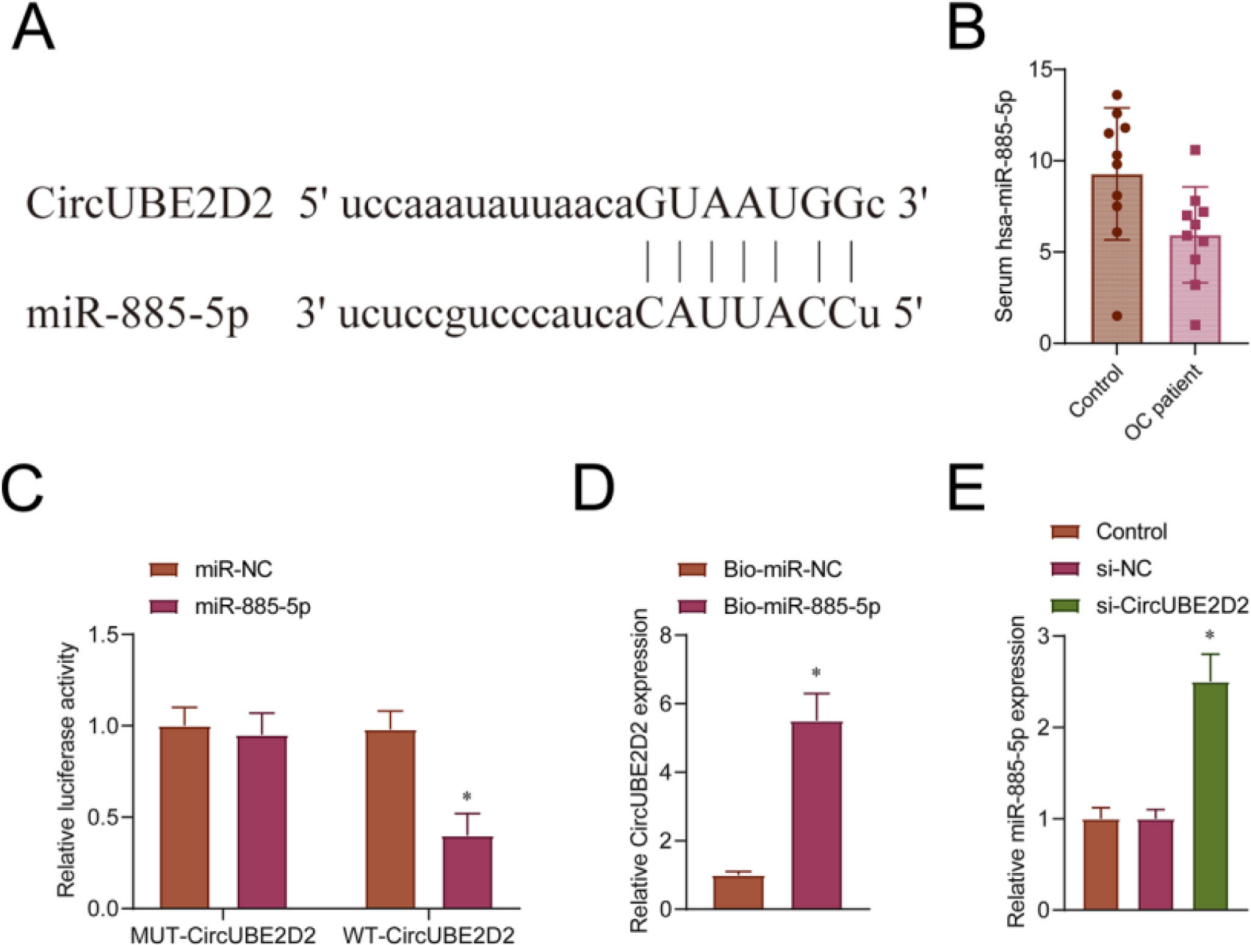
CircUBE2D2 serves as the ceRNA of miR-885-5p. (A) Schematic diagram of complementary nucleotide sequences of circUBE2D2 and miR-885-5p identified by starBase database. (B) GEO DataStes (https://www.ncbi.nlm.nih.gov/) obtained GSE216150 data to analyze miR-885-5p expression in OC patient's serum and normal serum. (C) Determination of luciferase activity. (D) RNA pull-down assay results. (E) RT-qPCR measurements of miR-885-5p expression in SKOV-3 cells (*p < 0.05).

One of the CircUBE2D2 targets, miR-885-5p, was screened from the starBase database. The predicted binding sites are shown in Figure 4A. Data from GSE216150 obtained from GEO DataStes showed that the expression of miR-885-5p in the serum of OC patients was down-regulated compared to the normal group (Fig. 4B). The circUBE2D2 fragment carrying the miR-885-5p binding sequence was cloned into the luciferase vector and mutated into the target region. In SKOV-3 cells, WT-CircUBE2D2 combined with miR-885-5p mimic induced a significant reduction in luciferase activity (Fig. 4C). At the mutation site of miR-885-5p (MUT-circUBE2D2), there was no reduction in luciferase activity (Fig. 4C). RNA pull-down analysis showed that circUBE2D2 enrichment was increased with Bio-miR-885-5p incubation (Fig. 4D). CircUBE2D2 knockdown caused miR-885-5p to be up-regulated (Fig. 4E).

### CircUBE2D2 regulates cell behaviors in vitro by targeting miR-885-5p

**Fig. 5:**
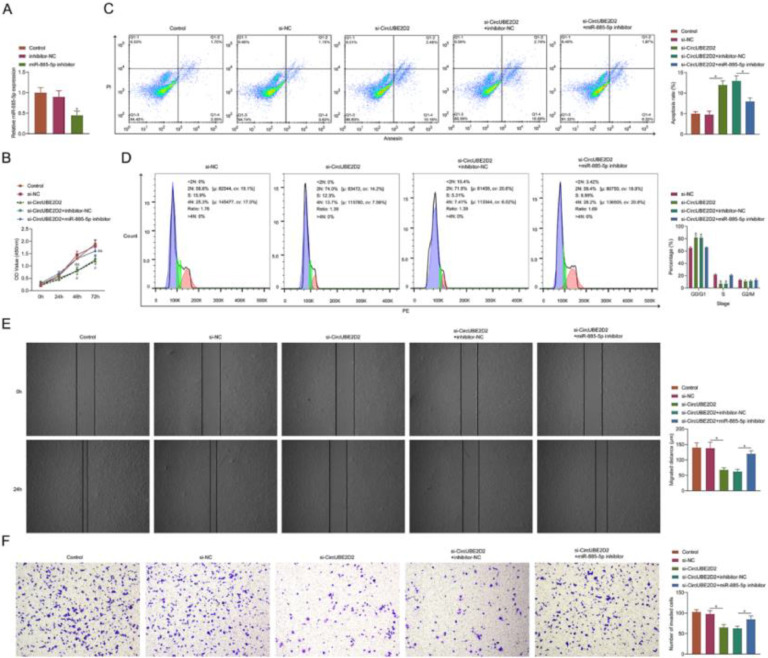
CircUBE2D2 regulates cell behavior *in vitro* by targeting miR-885-5p. (A) RT-qPCR measurements of miR-885-5p. (B) Determination of CCK-8 cell viability. (C‒D) Flow cytometry analysis of apoptosis and cell cycle. (E) Cell scratch migration assay. (F) Assay of Transwell invasion (×100; *p < 0.05).

The authors evaluated whether miR-885-5p is the molecular medium that circUBE2D2 functions in the OC progression. Transfection efficiency of miR-885-5p inhibitor was determined by RT-qPCR (Fig. 5A). The collected data reflected that lessening miR-885-5p eliminated the roles of si-UBE2D2-mediated anti-survival (Fig. 5B), pro-apoptosis and induction of cell cycle stasis in the G0/G1 phase (Fig. 5C‒D), anti-migration (Fig. 5E), and anti-invasion (Fig. 5F) in SKOV-3 cells.

### HMGB1 was directly inhibited by miR-885-5p in vitro

**Fig. 6:**
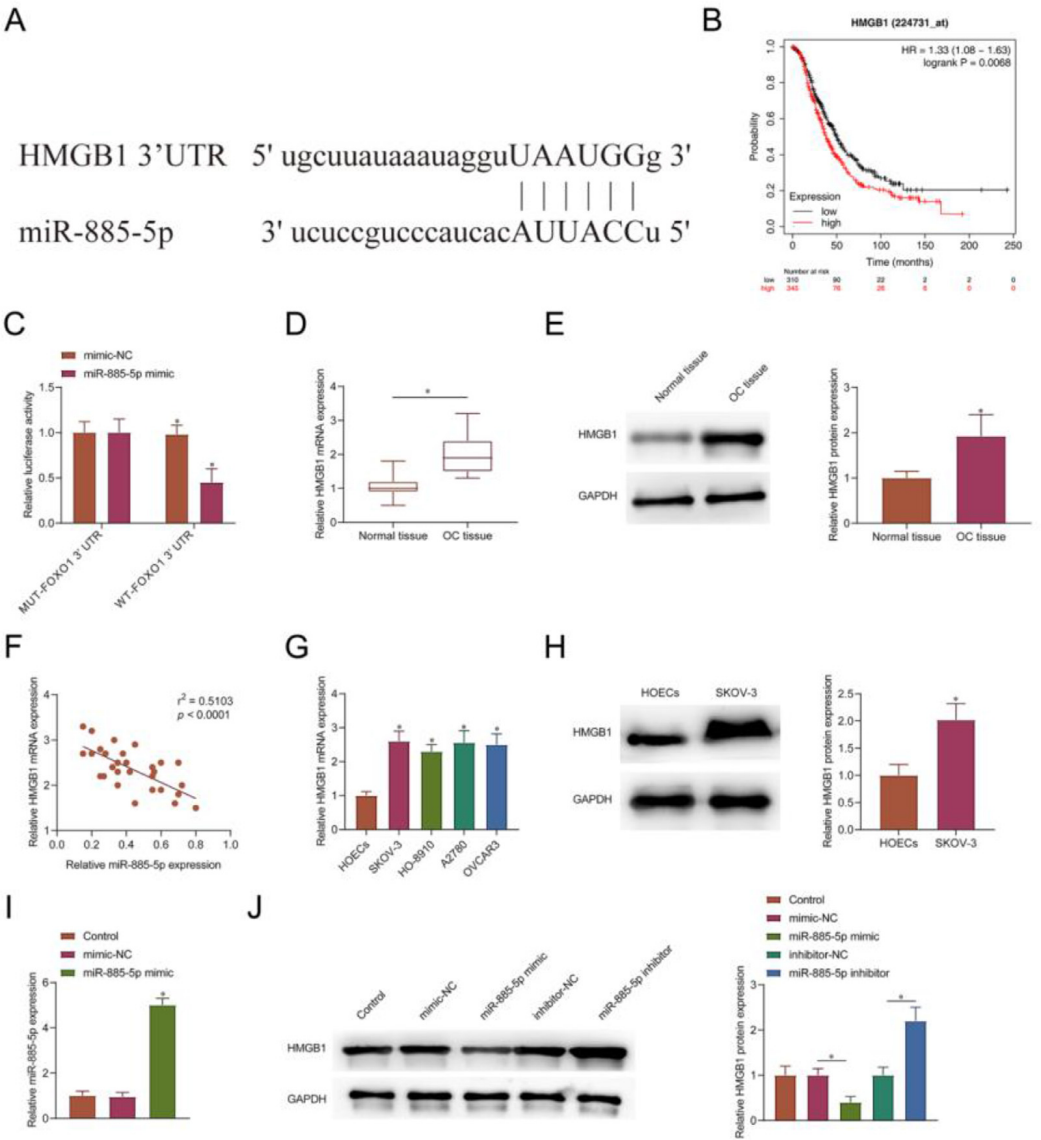
HMGB1 is directly inhibited by miR-885-5p. (A) Schematic diagram of miR-885-5p and HMGB1 complementary nucleotide sequences predicted by TargetScan database. (B) Kaplan Meier Plotter database (http://kmplot.com/analysis/index.php?p=background) analyzed the correlation between HMGB1 and OC patients' overall survival. (C) Luciferase activity in SKOV-3 cell. (D‒E) RT-qPCR and Western blot measurements of HMGB1 in OC tissues and matched normal tissues, and (F‒G) in OC cells and HOECs cells. (H) Spearman test to evaluate the correlation between HMGB1 mRNA expression and miR-885-5p levels in OC. (I) RT-qPCR measurements of miR-885-5p in SKOV-3 cells. (J) Western blot measurements of HMGB1 protein in SKOV-3 cells (*p < 0.05).

According to TargetScan, HMGB1 3′UTR contained complementary nucleotides of miR-885-5p (Fig. 6A). According to the Kaplan-Meier Plotter database, higher HMGB1 expression was associated with poorer prognosis in OC patients (Fig. 6B). In the presence of miR-885-5p mimics, WT-HMGB1 3′UTR resulted in a strong reduction in luciferase activity, and this effect was eliminated by mutations in the miR-885-5P complement sequence (MUT-HMGB1 3′UTR, Fig. 6C). In OC, HMGB1 levels were elevated (Fig. 6D‒E), and HMGB1 expression was negatively correlated with miR-885-5p levels (Fig. 6F). In addition, HMGB1 levels in OC cells were enhanced (Fig. 6G‒H). Transfection efficiency of miR-885-5p mimics was evaluated using RT-qPCR (Fig. 6I). As expected, miR-885-5p mimics repressed HMGB1 protein levels while silencing miR-885-5p forced HMGB1 protein levels (Fig. 6J).

### miR-885-5p regulates cell behavior in vitro by downregulating HMGB1

**Fig. 7:**
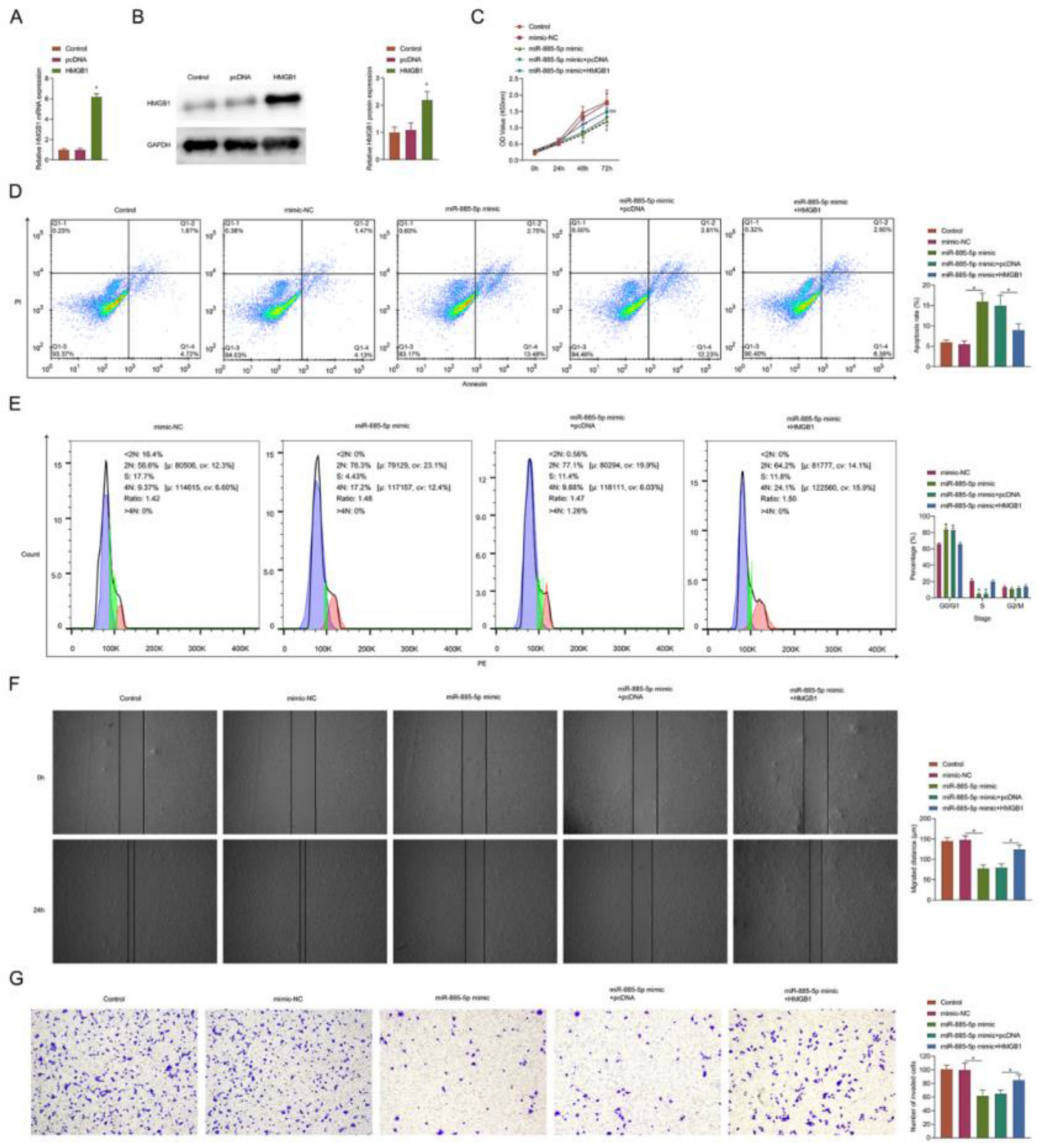
miR-885-5p induction regulates cell behavior by down-regulating HMGB1. (A‒B) RT-qPCR and Western blot measurements of HMGB1. (C) Determination of CCK-8 cell viability. (D‒E) Flow cytometry analysis of apoptosis and cell cycle. (F) Cell scratch migration assay. (G) Assay of Transwell invasion. (×100; *p < 0.05).

When HMGB1 overexpressed plasmid was transfected into SKOV-3 cells, HMGB1 mRNA and protein expressions were induced (Fig. 7A‒B). Elevating miR-885-5p significantly inhibited cell viability (Fig. 7C) promoted apoptosis and increased the proportion of G0/G1 phase cells (Fig. 7D‒E), and inhibited cell migration (Fig. 7F) and invasion rate (Fig. 7G). However, high expression of HMGB1 significantly saved these effects of miR-885-5p induction in SKOV-3 cells (Fig. 7C‒G).

### CircUBE2D2 regulates HMGB1 expression through miR-885-5p

**Fig. 8:**
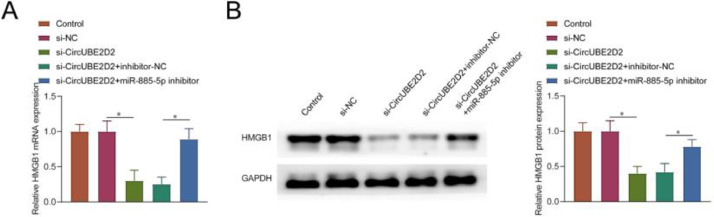
CircUBE2D2 regulates HMGB1 expression through miR-885-5p. (A‒B) RT-qPCR and Western blot measurements of HMGB1 in SKOV-3 cells (*p < 0.05).

Subsequently, the authors examined whether circUBE2D2 regulates HMGB1 expression in SKOV-3 cells. As expected, circUBE2D2 silence significantly down-regulated HMGB1 expression. However, the introduction of miR-885-5p inhibitor significantly reversed this effect (Fig. 8A‒B).

### CircUBE2D2 silence inhibits mouse tumor growth

**Fig. 9:**
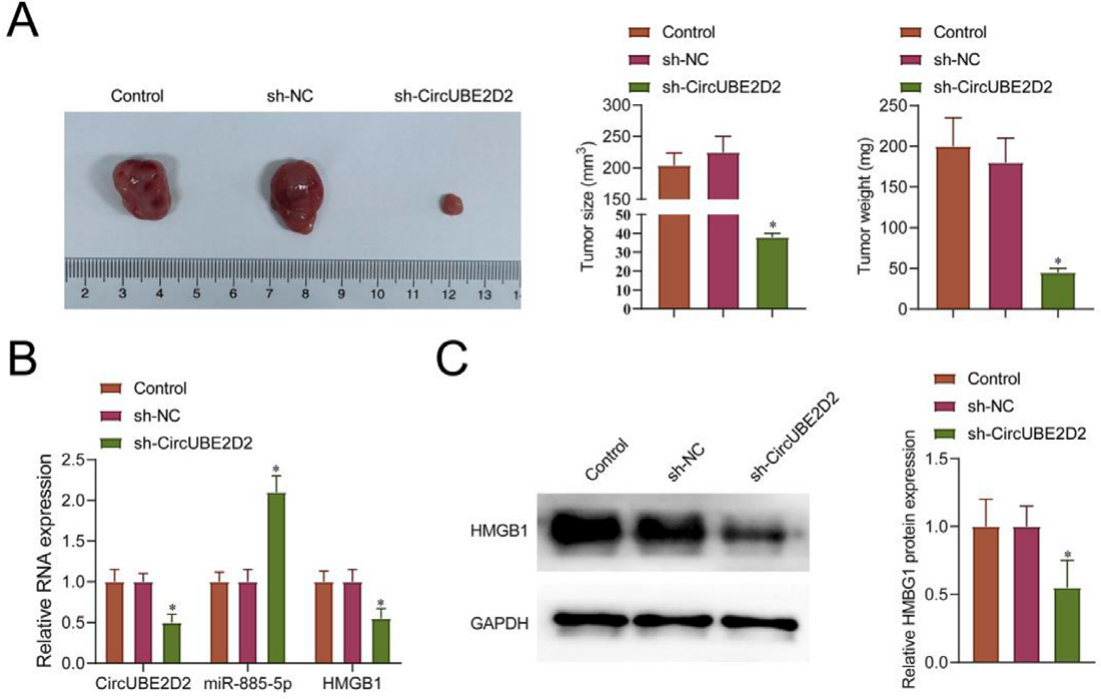
CircUBE2D2 suppresses tumor growth *in vivo*. (A) Tumor volume and weight. (B‒C) RT-qPCR or Western blot measurements of circUBE2D2, miR-885-5p, and HMGB1 in xenograft tumors (*p < 0.05).

sh-circUBE2D2 transfected or sh-NC transfected SKOV-3 cells were implanted in nude mice. The transduction of sh-circUBE2D2 significantly inhibited tumor growth (Fig. 9A). In tumors transfected with sh-sh-circUBE2D2, circUBE2D2 and HMGB1 levels were suppressed, while miR-885-5p were enhanced (Fig. 9B‒C).

## Discussion

In recent years, CircRNA has become a research hotspot due to its role in tumorigenesis and cancer progression.^24^ CircRNA regulates gene expression at the transcriptional and translational levels, directing DNA synthesis or gene rearrangement. CircRNA is an interaction between ceRNA and miRNA and mRNA is known as ceRNA crosstalk. CircRNA, as ceRNA, regulates gene expression, thereby inhibiting miRNA's degradation of mRNA. A large number of studies have confirmed that this mechanism is involved in regulating the biological behavior of various tumors, such as the migration and invasion of cancer cells. In this study, the authors demonstrated for the first time that CircUBE2D2 is a carcinogenic factor that regulates OC cell metastasis. CircUBE2D2 regulates the expression of HMGB1 by targeting miR-885-5p and promotes cancer progression.

As a novel gene regulatory factor, circRNA has been increasingly proven to play a role as an oncogene or tumor suppressor in the occurrence and development of cancer. For example, Circ-BNC2 is underexpressed in OC samples, and its overexpression can inhibit OC cell proliferation, migration, and invasion.^25^ Circ-NOLC1 is highly expressed in epithelial OC and is considered a tumor-promoting circRNA to promote the development of malignant behavior in OC cells.^26^ The present data suggest that the high expression of CircUBE2D2 in OC samples may act as a tumor-promoting factor that regulates the occurrence and progression of OC. The authors verified that CircUBE2D2 was highly expressed in OC tissues and cells by RT-qPCR. In addition, CircUBE2D2 was associated with cancer stage, lymphatic metastasis, and poor prognosis in OC patients. The authors then further demonstrated by CCK8 and flow cytometry, cell scratch test and Transwell that silencing CircUBE2D2 inhibits OC growth, proliferation, migration, and invasion and promotes apoptosis. These results all suggest that CircUBE2D2 is involved in metastasis and malignant progression of OC and can be used as a biomarker for the diagnosis and prognosis of OC.

Circular RNA localization is closely related to the sponge effect on miRNA.^27^ CircRNAs derived from exons are usually present in the cytoplasm. The authors predict by the CSCD database (http://gb.whu.edu.cn/CSCD) and nuclear separation experiments to determine the CircUBE2D2 subcellular localization. These experiments determined that CircUBE2D2 is primarily present in the cytoplasm of OC cells and showed that circular UBE2D2 is RNase R resistant. Subsequently, it was found that miR-885-5p was one of the targets of CircUBE2D2 by Starbase3.0 prediction. Subsequently, data from GSE216150 showed that serum miR-885-5p expression was down-regulated in OC patients compared to normal groups. In summary, it is speculated that miR-885-5p functions as a tumor suppressor RNA in the negative regulation of OC. The present findings are similar to previous findings regarding miR-885-5p in neuroblastoma and hepatocellular carcinoma. miR-885-5p was lowly expressed in neuroblastoma, and its strong expression had a tumor inhibition effect and interfered with cell cycle entry and cell survival.^28^ miR-885-5p was strongly down-regulated in hepatocellular carcinoma, and its induction inhibited tumor cell proliferation and migration.^19^ Interestingly, miR-885-5p is highly expressed in patients with early pancreatic cancer.^29^ However, no studies have confirmed whether miR-885-5p is regulated by CircUBE2D2 and thus involved in OC progression. The authors confirmed the targeting relationship between miR-885-5p and CircUBE2D2 by dual luciferase reporter assay. In addition, silencing CircUBE2D2 inhibited the progression of OC cells, but inhibiting the expression of miR-885-5p eliminated the inhibitory effect of CircUBE2D2 silencing on the proliferation, migration and invasion behavior of OC cells. These results suggest that CircUBE2D2 can target the expression of miR-885-5p to regulate the development and progression of OC. miRNAs inhibit mRNA translation and/or cleavage by interacting with mRNA, ultimately leading to the down-regulation of protein expression.^30^ Kaplan-Meier Plotter analysis showed that higher HMGB1 expression was associated with poor prognosis in OV patients. The present study confirmed that HMGB1 was highly expressed in OC and was a functional target of miR-885-5p in OC. HMGB1 is a nuclear DNA-binding protein ubiquitous in mammalian cells and involved in transcriptional regulation of gene expression.^31^ More and more evidence shows the involvement of HMGB1 in the transcription of cancer-related genes.^32^^,^^33^ HMGB1 is highly expressed in cancers, and down-regulation of HMGB1 represses tumor cells to proliferate.^34^^,^^35^ It has been reported that HMGB1 can promote cell proliferation and metastasis by regulating downstream RAGE/ERK,^36^ TNFR1/NF-Κb,^37^ and TLR4/HMGB1 ^38^ signaling pathways. Based on this, in order to explore CircUBE2D2 as the ceRNA of miR-885-5p, it can affect the progression of OC by regulating the expression of HMGB1. The authors showed for the first time that CircUBE2D2 acts as a regulator of HMGB1 expression through miR-885-5p. *In vitro* studies confirmed that silencing CircUBE2D2 significantly down-regulated HMGB1 expression, while miR-885-5p inhibitor reversed this result. miR-885-5p mimic inhibited the proliferation, migration, and invasion of OC cells, but overexpression of HMGB1 eliminated the role of miR-885-5p mimic in inhibiting the progression of OC cells. In addition, *in vivo* studies confirmed that lentivirus-mediated CircUBE2D2 silencing led to the increased miR-885-5p expression, down-regulated HMGB1 expression, and reduced tumor growth in xenografts.

However, there are some limitations to this study. The regulatory mechanism of CircRNA in malignant tumors is complex. Furthermore, circRNAs have been shown to function not only as competing ceRNAs, but also as protein scaffolds and regulators of transcription and splicing. This study did not include a comprehensive analysis of ovarian cancer patients and normal tissue samples, specifically examining the association between CircUBE2D2 expression and the tumor immune microenvironment; The molecular mechanism of CircUBE2D2 regulating the downstream pathway of HMGB1 is not clear. In subsequent studies, the authors will further analyze the relationship between CircRNA in OC tissues and the immune microenvironment or immune escape of OC tumors. At the same time, more molecular and animal studies will be conducted to explore the role of CircUBE2D2 in OC.

In this study, CircUBE2D2 was highly expressed in OC tissues and cells. Patients with high CircUBE2D2 expression had higher FIGO stage and cancer cell differentiation and were associated with poor prognosis. CircUBE2D2 can therefore be used as a biomarker for the diagnosis and treatment of OC. CircUBE2D2 was predicted as the ceRNA of miR-885-5p. Functionally, CircUBE2D2 silencing inhibited the proliferation, migration and invasion of OC cells and promoted apoptosis. Mechanistically, CircUBE2D2 acts as the ceRNA of miR-885-5p to regulate the expression of HMGB1, thus participating in the occurrence and progression of OC. The present data therefore enhance the understanding of circRNA biology, and these results highlight that CircUBE2D2 offers broad promise for the diagnosis and treatment of OC.

## Conflicts of interest

The authors declare no conflicts of interest.
